# Exploring the effects of culture conditions on Yapsin (*YPS*) gene expression in *Nakaseomyces glabratus*

**DOI:** 10.1515/biol-2022-0995

**Published:** 2024-11-26

**Authors:** Aneta Bednarek, Agnieszka Kabut, Maria Rapala-Kozik, Dorota Satala

**Affiliations:** Department of Comparative Biochemistry and Bioanalytics, Faculty of Biochemistry, Biophysics and Biotechnology, Jagiellonian University, Gronostajowa 7, 30-387, Kraków, Poland; Doctoral School of Exact and Natural Sciences, Faculty of Biochemistry, Biophysics and Biotechnology, Jagiellonian University, Gronostajowa 7, 30-387, Kraków, Poland

**Keywords:** *Nakaseomyces glabratus*, *YPS* gene expression, fungal pathogenicity, protease regulation, host niche adaptation

## Abstract

*Nakaseomyces glabratus*, previously known as *Candida glabrata*, has the great potential to cause systemic fungal infections despite its similarity to baker’s yeast. Its pathogenicity is attributed to the production of numerous virulence factors, among which the *YPS* genes (*YPS1*–*YPS11*) encoding aspartyl proteases have yet to be sufficiently characterized, and limited studies suggest their involvement in cellular homeostasis. The study’s novelty is an investigation of the role of *YPS* in *N. glabratus’s* ability to adapt to different host environments. For this purpose, we isolated RNA from *N. glabratus* cells grown in both host niche-mimicking culture media, such as artificial saliva (AS) and vagina-simulating media (VS), as well as standard yeast media (RPMI 1640 and YPDA). We then performed quantitative real-time PCR to evaluate *YPS* gene expression at different growth phases. At the early logarithmic phase, we observed a general increase in the expression levels of *YPS* genes; however, at the stationary phase, high expression levels were maintained for *YPS7* in RPMI 1640 and YPDA media and *YPS6* in RPMI 1640 and AS media. In addition, although the VS medium does not promote the proliferation of *N. glabratus*, the yeast can survive in an acidic environment, and the significantly overexpressed gene is *YPS7*. These findings underscore the significant modulation of *N. glabratus YPS* gene expression in response to external environmental conditions. This research provides insights into the molecular basis of *N. glabratus* pathogenicity and highlights new potential targets for antifungal therapy.

## Graphical abstract

**Figure:**
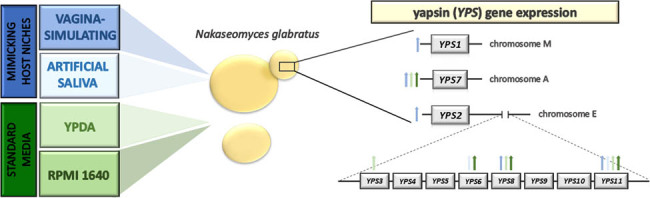

## 1 Introduction

*Nakaseomyces glabratus* – previously identified as *Candida glabrata* – exhibits high infectious potential despite its close phylogenetic relationship with baker’s yeast. It is the second most common pathogen, following *Candida albicans*, and is responsible for many mucosal and systemic candidiasis cases, particularly among immunocompromised individuals [1,2]. In recent decades, the increasing incidence of *N. glabratus* infections and its resistance to fluconazole has posed a significant challenge for medical management [3,4,5,6].

Fungal pathogens use a wide range of molecules, enabling them to invade deep into host tissues, evade defense mechanisms and immune surveillance, and spread to distant niches in the host, where they begin colonization [7,8,9]. This repertoire of molecules includes proteins that are expressed on the cell surface and secreted outside the cell [10,11,12,13,14]. In the case of the primary fungal pathogen *C. albicans*, it has been shown that one such group of moleclues attributed to pathogenic potential is proteases, specifically the secreted aspartyl proteases family (Sap), which has at least ten identified members [15,16]. These proteases play critical roles in the infection process, including nutrient acquisition through the degradation of host molecules, facilitating adhesion and tissue invasion by disrupting host cell membranes, and evading host immune defenses by degrading host cells and immune molecules [17,18,19,20,21,22,23,24,25,26,27,28]. Interestingly, *N. glabratus* is a unique species that does not produce the typical candidal extracellular aspartyl proteases belonging to the Sap family. Instead, it displays on its cell surface a poorly characterized group of proteases from a family known as yapsin (Yps) [22,29]. The *N. glabratus* genome contains at least 11 genes encoding Yps, most of which are likely covalently attached to the cell wall via glycosylphosphatidylinositol anchors located at the C-terminus of the proteins [30].

In the case of *C. albicans*, *SAP* expression is modulated by various environmental factors, including pH changes, and by interactions with the host, e.g., adhesion to vaginal epithelial cells or interactions with serum components. These results suggest that *C. albicans* can fine-tune its virulence factors through sophisticated mechanisms in response to specific host niches [26,31,32,33,34,35,36]. The data on changes in *N. glabratus YPS* expression is limited to a few reports that demonstrated that upon internalization by neutrophils, the fungus overexpressed *YPS1*, *YPS2*, *YPS4*-6, and *YPS8-11*, whereas macrophage-associated yeasts overexpress *YPS2*, *YPS4*, *YPS5*, and *YPS8*-11 [37,38]. Furthermore, Ferrari et al. [39] highlighted the significance of Yps in virulence, indicating that strains of *N. glabratus* that are resistant to azoles and exhibit mitochondrial dysfunction (BPY41) overexpress genes encoding these proteases.

The primary aim of this study was to investigate the regulatory mechanisms controlling *YPS* gene expression in *N. glabratus* under diverse environmental conditions to elucidate how these proteases contribute to the pathogen’s adaptation and survival within different host niches. By examining *YPS* gene expression not only in standard yeast culture media (RPMI 1640 and YPDA) but also in host-mimicking environments such as artificial saliva (AS) and vagina-simulating media (VS), we sought to identify specific proteases that respond to variations in pH and nutrient availability. The results of this study are intended to deepen our understanding of the adaptive strategies employed by *N. glabratus* and to highlight potential targets for therapeutic intervention and diagnostics in fungal infections.

## 2 Materials and methods

### 2.1 Yeast strains and culture conditions

Cells of the *N. glabratus* strain CBS138 (ATCC^®^ 2001™) purchased from the American Type Culture Collection (Manassas, VA, USA) were cultured in yeast peptone dextrose (YPD) medium (1% yeast extract, 2% soy peptone, and 2% glucose, pH 6.0; Sigma, St. Louis, MO, USA) for 16 h at 30°C with shaking at 170 rpm. To measure the optical density (OD), 20 µl of the culture was inoculated into 20 ml of different growth media and cultured for approximately 25 h at 37°C and 170 rpm. Then, OD at 600 nm was measured every hour using a Synergy H1 microplate reader (BioTek Instruments, Winooski, VT, USA); however, in cases where the measured OD exceeded 1, samples were diluted 10-fold to ensure accurate measurements and avoid signal saturation. This measurement allowed us to track the exponential growth phase and assess potential growth inhibition in different media, providing crucial data on how varying conditions impact the proliferation of *N. glabratus*. Based on the observed logarithmic growth of cells over time, the values of the coefficient-specific growth rate (*µ*_max_) were calculated using the formula *μ*_max_  =  (ln OD_max_ – ln OD_min_)/Δ*t*_log_ [40]. The media used in the experiments is the Roswell Park Memorial Institute (RPMI 1640) defined media (PAA Laboratories GmbH, Pasching, Austria), YPDA media – YPD buffered media with a reduced content of animal-derived peptone (0.1% yeast extract, 0.2% animal peptone, and 2% glucose, pH 7.0) supplemented with 10 mM NaH_2_PO_4_, and media mimicking the vaginal microenvironment (VS, pH 4.2), prepared according to the procedure of Moosa et al. [41], and a media consisting of chemically defined AS (pH 7.0), prepared according to the procedure described in the work of Wong and Sissons [42]. Cells intended for RNA isolation were collected at two points of growth – the early logarithmic phase (4 h) and the stationary phase (18 h). Exceptionally, in the case of the VS medium, 50 times more cells were used for inoculation, which, according to the OD measurement, was approximately 7.5 × 10^8^ cells per 20 ml of medium. In this medium, the material for RNA isolation was collected after 18 h.

### 2.2 Cell viability

Cell integrity was examined using Sytox™ Green nucleic acid stain (Thermo Fisher Scientific, Waltham, MA, USA). For this purpose, 1 × 10^5^ *N. glabratus* cells cultured in VS and YPD media for 18 h were placed in the wells of a glass-like microplate (CellVis, Mountain View, CA, USA). After adding the dye, they were imaged using an Olympus IX73 microscope (Olympus, Tokyo, Japan). In addition, cell metabolic activity was measured using an assay based on the quantitation of ATP present in the culture. To each well of a 384-well polypropylene microplate (Greiner, Kremsmünster, Austria), 10^4^ *N. glabratus* cells cultured in YPD or VS media were added to a final volume of 25 µl of PBS. Then, starting the bioluminescence reaction, 50 µl of the BacTiter-Glo™ reagent (Promega, Madison, WI, USA) was added to each well according to the manufacturer’s instructions. After 15 min of incubation at room temperature, the luminescence level was measured using a Synergy H1 microplate reader (BioTek Instruments). To determine the CFUs (colony-forming units), *N. glabratus* colonies were counted after the same number of cells were seeded onto agar plates and incubated overnight at 37°C.

### 2.3 Isolation and purification of RNA

After overnight culture of *N. glabratus* grown in the YPD medium, the cells were transferred with appropriate media and cultured at 37°C with shaking at 170 rpm. After the specified times (4 h or 18 h), the cells were centrifuged for 6 min at 2,500 × *g*, followed by washing three times with PBS. The cells were centrifuged at 5,000 × *g*, the filtrate was removed, and the pellet was frozen in liquid nitrogen. Once thawed, the cells were transferred to a tube containing 0.4 ml of glass beads (25–600 μm, Sigma Aldrich, St. Louis, MO, USA) and 0.8 ml of Tri Reagent (Sigma Aldrich). The suspension was then shaken using a FastPrep Precellys Evolution (Bertin Technology, Montigny-le-Bretonneu, France) for 2 cycles at 6 rpm for 45 s with a 2-min break on ice between cycles. In the next step, 160 µl of chloroform was added to the lysate, vortexed for 15 s, and incubated at 4°C for 30 min. After incubation, the suspension was centrifuged at 10,000 × *g* at 4°C for 15 min. The filtrate (phase with nucleic acids) was transferred to a new tube, and isopropanol was added to a volume equal to the mixture, and the mixture was incubated overnight at −20°C. The next day, the mixture was centrifuged at 10,000 × *g* for 15 min at 4°C, the filtrate was removed, and the pellet was resuspended in 70% ethanol, incubated for 1 min, vortexed, and then centrifuged under the same conditions. This step was repeated until the pellet was thoroughly dried and resuspended in RNase-free water. The whole pellet was incubated at 56°C for 5 min. RNA purity was verified using a NanoDrop spectrophotometer (Thermo Fisher Scientific), with A260/A280 ratios between 1.8 and 2.0 considered indicative of high purity. Additionally, RNA integrity was confirmed electrophoretically on a 1.5% agarose gel stained with ethidium bromide using the RiboRuler High Range RNA Ladder (Thermo Scientific) (Figure S1 in supplementary material), ensuring the absence of degradation prior to downstream qPCR analyses. These steps were critical to guarantee the accuracy and reproducibility of gene expression results obtained in subsequent experiments.

### 2.4 Real-time PCR (qPCR)

The level of *YPS1*–*11* transcripts was determined by real-time PCR (qPCR). For this purpose, information from isolated RNA was transcribed into cDNA via reverse transcription. In the first step of this reaction, 2 µg of total RNA was resuspended in RNase-free water supplemented with 0.5 g of oligo(dT)18 primer (Genomed, Warszawa, Poland), heated at 70°C for 5 min, and then cooled to 4°C. In the second step, a reaction mixture containing 200 U of MLV reverse transcriptase (Moloney murine leukemia virus reverse transcriptase; Promega, Madison, WI, USA) was added to the sample, and the PCR was run in a C1000 Touch Thermal Cycler (Bio-Rad, Hercules, CA, USA) for 1 h at 42°C and 10 min at 70°C. For qPCR, 4 times-diluted cDNA was used and applied in triplicate (2 µl per well) to a MicroAmp Fast 96-well reaction plate (Thermo Fisher Scientific), followed by the addition of 8 µl of the reaction mixture, which was prepared according to instructions of the SYBR™ qRT‒PCR Kit (A&A Biotechnology, Gdynia, Poland), which provides high sensitivity for detecting gene expression changes. In addition, a negative control for each gene was performed, in which 2 µl of RNase-free water was added to the well of the plate instead of the cDNA matrix. The reaction was carried out on a QuantStudio™ 3 Real-Time PCR System (Thermo Fisher Scientific) under the following conditions: initial denaturation at 95°C for 5 min and 40 cycles of denaturation at 95°C for 15 s, primer annealing at 58°C for 15 s, and extension at 72°C for 20 s for genes *YPS1*–*10*; for *YPS11,* the only difference was the primer annealing temperature of 56°C. The relative quantification of *YPS* genes was performed using *ACT1,* commonly used as a housekeeping gene [43,44]. The primers for each gene were obtained from Genomed (Warszawa, Poland), and their sequences were obtained from the work of Kaur et al. [37]. Melting curve analysis was performed to evaluate the quality of the qPCR results, and relative gene expression levels were analyzed using the ∆∆Cq method [45], where the cell culture in the YPD medium for 4 and 18 h, respectively, was used as the reference growing conditions. The experiments were performed in triplicate with two biological replicates.

## 3 Results

In this study, three primary media commonly used for culturing *S. cerevisiae* and *Candida* spp. cells were employed: YPD (pH 6.0), RPMI 1640 (pH 7.4), and YPD-buffered medium with a reduced content of animal-derived peptone (YPDA, pH 7.0). The YPD medium comprises yeast extract, peptone, and dextrose, where the yeast extract contains essential vitamins and micronutrients, peptone is a source of amino acids and peptides, and dextrose acts as a carbon source. This composition optimizes the growth and proliferation of *Candida* spp. Conversely, RPMI 1640 is primarily used in studies investigating *Candida* susceptibility to antibiotics and biofilm formation [46]. The YPDA medium, distinguished by its reduced nitrogen content, predominantly induces the production of proteases [47]; however, at 37°C, it also promotes the generation of filamentous forms of *Candida* spp. Although *N. glabratus* cannot produce filamentous cells, which are commonly considered the most virulent form of other *Candida* species, significant changes in the proteome composition have been demonstrated to occur on the surface of the yeast depending on the culture medium used [48], highlighting the validity of using different types of media in our study. Two types of media with varying compositions and pH levels were used to simulate different potential sites of infection – AS (pH 7.0) to mimic the oral cavity [42] and VS (pH 4.2) to represent the vaginal microenvironment [41].

**Figure 1:**
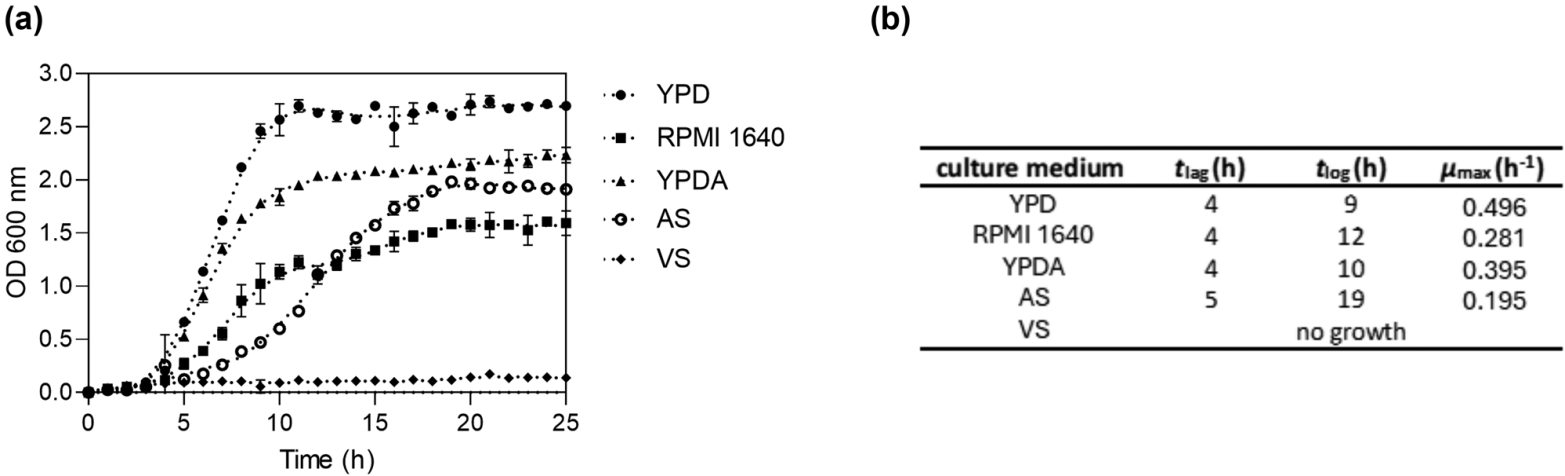
Analysis of *N. glabratus* growth in different culture media. (a) Growth curves of *N. glabratus* based on OD measurements taken hourly over 25 h. Values are presented as the mean ± SD from a representative experiment conducted in triplicate. (b) Growth parameters of *N. glabratus*, calculated from the OD data.

**Figure 2:**
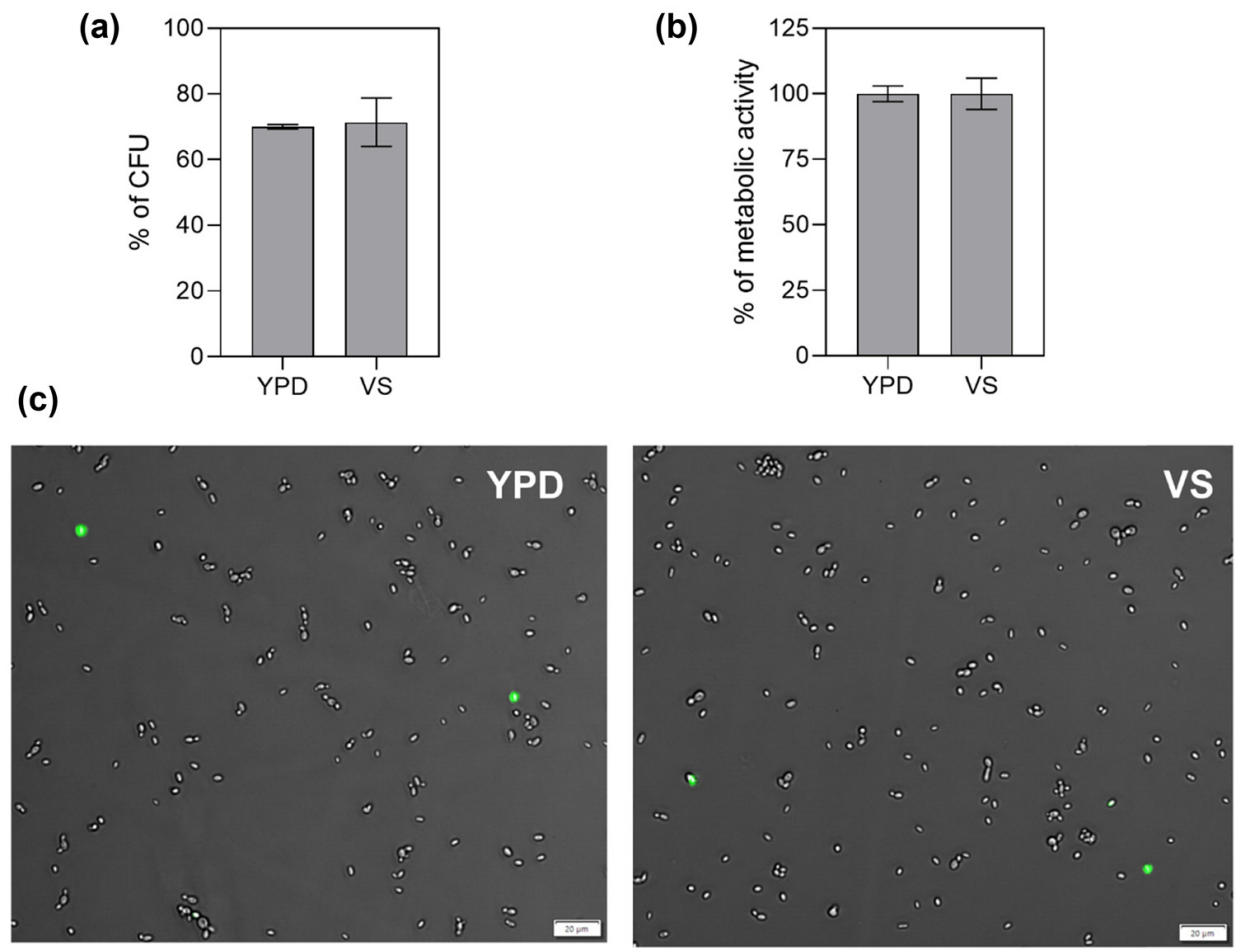
Survival of *N. glabratus* cells in the VS medium. *N. glabratus* cells (1 × 10^7^) were cultured for 18 h in the VS medium at 37°C, and then (a) CFU assays were performed in which 100 cells per plate were seeded based on OD measurements, corresponding to 100% CFU in the graph; (b) cell viability was assessed based on the amount of ATP present in 1 × 10^4^ cells per well of the microplate; and (c) cells were stained with SYTOX Green and imaged on an Olympus IX73 microscope as an overlay of bright field and FITC (BF/FITC). An equal number of *N. glabratus* cells cultured for 18 h in a YPD medium was used as a control for each analysis.

In the initial phase of our research, we conducted OD measurements to quantify the growth rate (*μ*_max_) of *N. glabratus* cultures (Figure 1). These measurements indicate that *N. glabratus* exhibits variable growth rates in response to different media compositions and environmental factors. In RPMI 1640 and YPDA media, the growth rate of *N. glabratus* was reduced compared to YPD (*μ*_max_ = 0.281, 0.395, and 0.496, respectively), as evidenced by a slower progression through the exponential phase. Despite this, the overall sigmoidal growth pattern was preserved. These findings suggest that while the nutrient composition and pH modulate growth dynamics, *N. glabratus* can adapt and maintain proliferation across diverse environments. In AS media, we showed delayed growth initiation (*μ*_max_ = 0.195) but eventually reached OD levels comparable to those of YPD media, indicating that although this medium may have limitations in supporting immediate growth, it ultimately sustains yeast proliferation to a similar degree. We recorded the lowest OD values in the VS medium, indicating an unfavorable environment for yeast growth or a potential inhibitory effect on *N. glabratus* proliferation. These results were unexpected, given that previous studies identified *N. glabratus* as one of the main pathogens isolated in cases of vaginal infection [49,50]. Therefore, we decided to conduct further analyses. Based on the CFU assay, microbial metabolic activity assay, and SYTOX Green staining revealing dead cells, we showed that although media-simulating vaginal conditions do not promote yeast cell proliferation, *N. glabratus* can survive in such environments (Figure 2). Thus, we decided not to exclude this medium from further analyses, raising the question of whether proteases play key roles in yeast survival under low-pH culture conditions.

**Figure 3:**
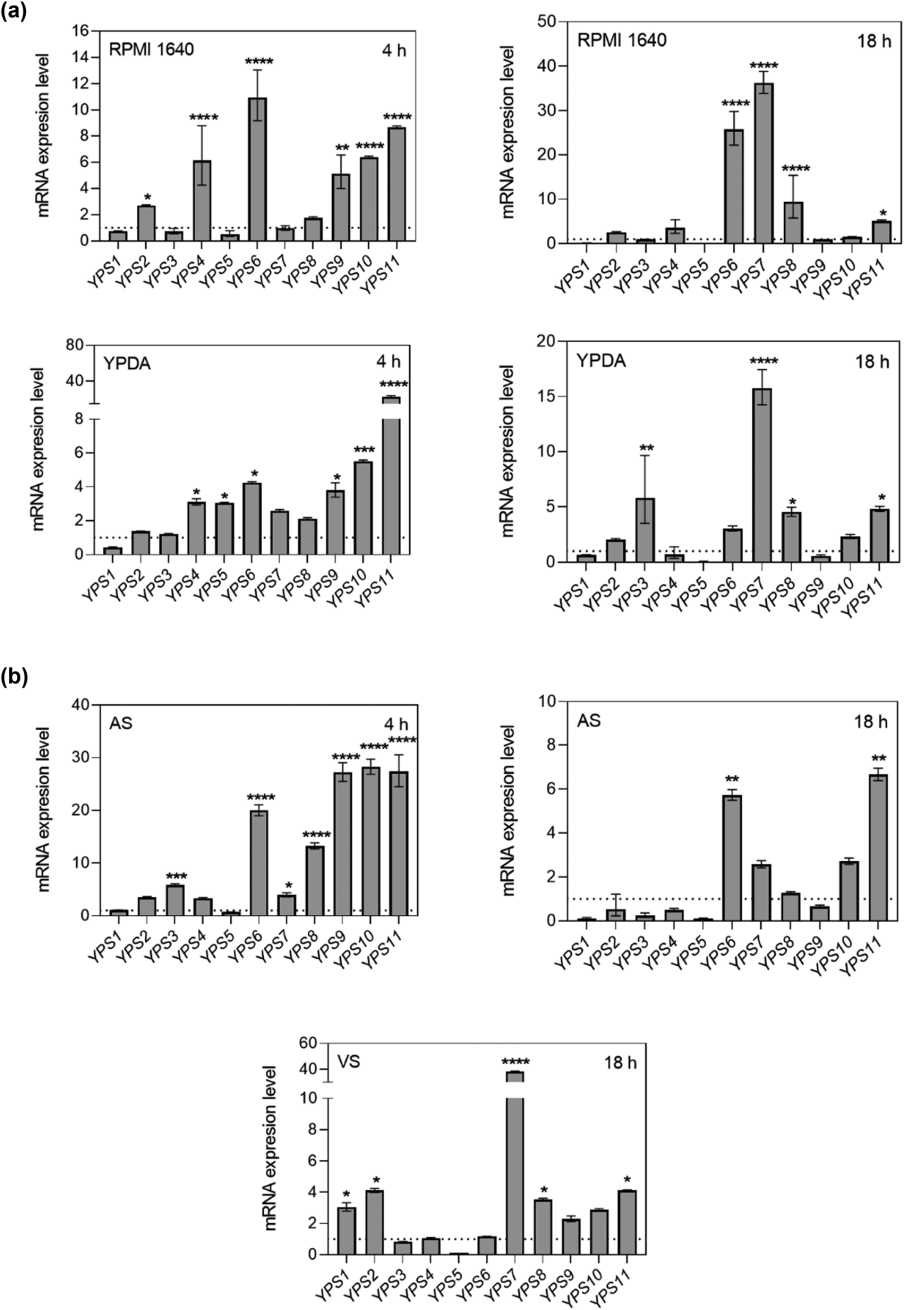
Changes in the expression of *N. glabratus* genes encoding *YPS* in response to changing culture conditions. Bars represent fungal gene expression levels in *N. glabratus* cells cultured in standard media (4 h and 18 h in RPMI 1640 and YPDA) or (b) in media mimicking host niches (4 h and 18 h of culture in the AS medium and 18 h in the VS medium). Gene expression levels were calculated by normalizing expression to that of *ATC1* and determined using the 2^−ΔΔCT^ method. The results were normalized to the YPD medium, in which the expression of individual *YPS* genes was set to 1 (as indicated by the dashed line in the graph). The bars indicate representative results of two independent experiments as the mean ± SD. The statistical significance of the reference was determined by one-way ANOVA with Dunnett’s multiple comparison test using GraphPad Prism software and is marked with *p  <  0.05, **p  <  0.01, ***p  <  0.001, ****p  <  0.0001.

In recent years, several advanced techniques for gene expression analysis have emerged, the most popular of which are RNA sequencing (RNA-seq) and microarrays. These methods enable high-throughput analyses determining the levels of thousands of genes per sample, which requires advanced bioinformatics analysis [51,52]. A significant advantage of RNA-seq is the ability to identify known and new transcriptomes [51], while microarrays, widely used in clinical studies, are limited in identifying predefined genes for which it is necessary to design appropriate probes [51,53]. Since the choice of the method is closely related to the study’s aim, and in our case, it was to investigate changes in the expression of a specific group of genes, we decided to perform analyses using the real-time PCR method. Due to its high sensitivity, which enables the detection of low-abundance transcripts, rapid measurements, accurate results, and relatively easy analysis, this method remains irreplaceable in basic research. Our analyses were performed on RNA isolated from different culture conditions in two growth phases – the early logarithmic phase (4 h) and the stationary phase (18 h), using cells cultured for analogous times in a YPD medium as a control. Using media with different compositions and pH values ranging from 4.2 to 7.4 provided insights into the regulatory mechanisms involved under different environmental stimuli (Figure 3). As shown, culturing for 4 h in RPMI 1640 and YPDA media leads to an increase in the expression of several *YPS* genes, namely *YPS4*, *YPS6*, and *YPS9*-*11*. Remarkably, the *YPS7* transcript level significantly increased by more than 30-fold in the RPMI 1640 medium and 15-fold in the YPDA medium after 18 h of culture. Furthermore, pronounced upregulation of *YPS6* (more than 20-fold) was observed in the RPMI 1640 medium, which coincided with a decrease in the expression of *YPS1* and *YPS5*. In turn, analysis of changes in gene expression after culturing in media that mimic the host niche revealed an increase of more than 20-fold in the expression of *YPS6* and *YPS8*–*YPS11* after 4 h in the AS medium. Extending the culture period under these conditions resulted in elevated *YPS6* and *YPS11* transcript levels; however, the increase did not exceed five-fold. Simultaneously, decreased expression of *YPS1*–*5* and *YPS9* was observed. In the case of the VS medium, which has a pH of 4.2, *YPS7* plays a predominant role, with a recorded increase in expression exceeding 35-fold. The only gene that exhibited a significant reduction in expression under these conditions was *YPS5*. Interestingly, a three-fold increase in *YPS1* expression was observed, in contrast with reduced transcript levels after 18 h in other media. The *YPS3*, *YPS4*, and *YPS6* genes are insensitive to acidic environments.

## 4 Discussion

The mechanisms of adaptation and virulence of *N. glabratus*, including its resistance to antifungal drugs and ability to survive in the host, underscore its significance in clinical settings and the necessity for ongoing research into its pathogenicity, which remains insufficiently understood [54,55,56,57]. Surprisingly, despite the widely described role of Saps in the pathogenesis of *C. albicans* [15,17,22,58–61] and its potential as components of anti-*Candida* vaccines [62–68], the functions of Yps are poorly understood. To date, *N. glabratus* proteases have not been isolated or characterized, indicating a gap in our understanding of the virulence strategies of this organism.

*YPS1*–*11* are distributed across three different chromosomes. *YPS1* is located on chromosome M, *YPS7* is located on chromosome A, and *YPS2* and a cluster unique to *N. glabratus,* including eight genes – *YPS3*–*6* and *YPS8*–*11* – are located on chromosome E [37]. To date, the main role of Yps has been their involvement in maintaining cellular homeostasis, including the cell wall structure and composition, vacuole integrity, pH and glucose homeostasis, secretome regulation, and stress resistance [37,69–71]. Interestingly, Yps are also essential for brain tissue colonization and biofilm formation on abiotic surfaces [69]. Miyazaki et al. [72] demonstrated the key role of *YPS1* in cell growth and cell wall integrity, especially under thermal stress conditions. Furthermore, the expression of *N. glabratus YPS1* under its native promoter was sufficient to rectify the cell wall defects observed in the *S. cerevisiae YPS1* mutant, suggesting that *YPS1* plays a role in maintaining glucan homeostasis [73]. Using mutants with deletions of one or more *YPS*-encoding genes, Yps1 was identified as an essential protein for survival under low pH conditions and menadione-induced oxidative stress [30,74]. This requirement is attributed to its impact on the ATPase activity of the plasma membrane proton pump Pma1 and the NADH:quinone oxidoreductase activity of the flavodoxin-like protein Pst2, which in turn leads to increased production of reactive oxygen species [30,74]. Consistent with these reports, in our analyses for the VS medium at pH 4.2, we also observed an increase in *YPS1* expression; however, under these conditions, the most significant increase in *YPS7* expression was observed. Interestingly, Cortés-Acosta et al. [75] demonstrated the genetic diversity of *YPS* genes in clinical *N. glabratus* strains. Despite the high conservation of the *YPS1* and *YPS7* genes across these strains, their regulatory regions exhibited significant polymorphisms characterized by diverse arrays of transcription factor-binding sites (TFBSs). A detailed analysis of eight clinical strains cultured under various environmental conditions – nitrogen and carbon starvation, cell wall stress, osmotic stress, acid stress, and thermotolerance – revealed substantial differences in the expression of *YPS1* and *YPS7*, ranging from underexpression to overexpression, among the strains tested [75]. Moreover, studies using mutant strains of *N. glabratus* with deletions in genes encoding *YPS*, particularly *YPS1* and *YPS7*, underscore their crucial role in pathogenesis, including colonization, dissemination, and maintenance within the kidneys, liver, and spleen, in a murine model of systemic infection [69].

Although our research revealed high variability in the expression of *YPS* genes in the cluster, especially *YPS6*, the current literature exhibited a noticeable gap regarding their potential function. A cluster-deleted mutant study showed that all eight genes were overexpressed upon contact with the murine macrophage line J774A.1, with *YPS3* and *YPS6* also overexpressed in the culture medium itself [37]. Deleting the *YPS* cluster alone did not induce phenotypic changes in disseminated infection models or macrophage-based assays. However, strains lacking *YPS1* or both *YPS1* and *YPS7* exhibited an apparent additive phenotype when the *YPS* cluster was deleted [37]. This evidence implies that the primary role of the *YPS* cluster is to interact with the host, and its functions may coincide with those of other *YPS* genes, especially *YPS1* [37]. These findings are supported by experiments conducted using the THP-1 line, in which the disruption of eight genes in the *YPS* cluster adversely affected the intracellular growth and viability of *YPS7* and *YPS1* mutants, respectively [69].

In our analyses, both under conditions mimicking the host environment and prolonged cultivation in standard media, the increased expression of *YPS6* was notable. Unfortunately, conclusions about its potential function based on similarities are difficult because, according to the Candida Genome Database, the protein structurally shares only 24% sequence homology with the Sap5 protein of *C. albicans*. Furthermore, the Yps6 protein lacks the RGD/KGD amino acid sequences identified as integrin-binding fragments in Sap5 [76]. Moreover, other proteins encoded within the same cluster exhibit structural similarities to *Candida parapsilosis* Sapp1 and *Candida tropicalis* Sapt, with sequence homology ranging from 30 to 34% (based on data in the Candida Genome Database). In a study of *C. parapsilosis*, the enzyme Sapp1 was associated with the cell wall, suggesting that its functionality may be similar to that of the enzymes Sap9 and Sap10 in *C. albicans* [77,78]. To date, these enzymes have been suggested to play a role in modulating the host inflammatory response. Namely, macrophages exposed to strains lacking *SAPP1–SAPP3* secrete lower levels of interleukins IL-1β and IL-6 than those exposed to wild-type strains [79]. Moreover, they induce the influx of neutrophils by modulating the production of chemokines [79]. From the perspective of *N. glabratus*, which shows a particular ability to survive in human macrophages, it is worth noting that Sapp1 and Sapp2 can inhibit phagosome–lysosome fusion, promoting the yeast survival in human immune cells. This may indicate similar functions of *N. glabratus* proteases and *C. parapsilosis* [79]. In the context of Sapt, recent discoveries have revealed the proteolytic effect of this proteases on C-type lectins, likely disrupting the activation of the lectin complement pathway [80].

Proteases from many microorganisms are used in various industries, such as pharmaceuticals, biotechnology, meat, dairy, and bakery production [81–83]. Although our analyses provide preliminary results to understand the regulation of YPS genes and their role in pathogenesis, they may find applications in the biotechnology industry. Using proteases as potential biomarkers of infection will enable early detection of the pathogen and rapid, targeted medical intervention. Due to the uniqueness of the *YPS* family, identifying proteases expression may be especially interesting in the case of mixed infections, which are particularly difficult to treat. Moreover, our results may find application in the pharmaceutical industry, indicating the direction of designing new drugs and therapies based on inhibition of the function of these enzymes.

## 5 Conclusion

In conclusion, this study provides novel insights into the dynamic regulation of *YPS* gene expression in *N. glabratus* under varying environmental conditions, particularly in host-simulating media. Our research highlights the critical roles of *YPS6* and *YPS7* in fungal adaptation to acidic and nutrient-limited environments, suggesting that these proteases may be key factors in the pathogen’s survival and virulence. Future studies should focus on understanding the direct functions of individual *YPS* proteases in disrupting host homeostasis. For this purpose, it would be necessary to isolate and purify native proteins and investigate their involvement in the evasion of the host immune response and tissue damage. Then, extending these studies to *in vivo* models will significantly deepen the understanding of *N. glabratus* pathogenic mechanisms and open new avenues for therapeutic interventions targeting *YPS* proteases.

## Supplementary Material

Supplementary Figure
