# Supplementary material for: Exploring the effects of culture conditions on Yapsin (YPS) gene expression in Nakaseomyces glabratus
Source: Open Life Sci. 2024 Nov 26;19(1):20220995. doi: 10.1515/biol-2022-0995 (PMC11627043; doi:10.1515/biol-2022-0995)
Supplement: Supplementary Figure [file biol-2022-0995-sm.pdf]

Supplementary material

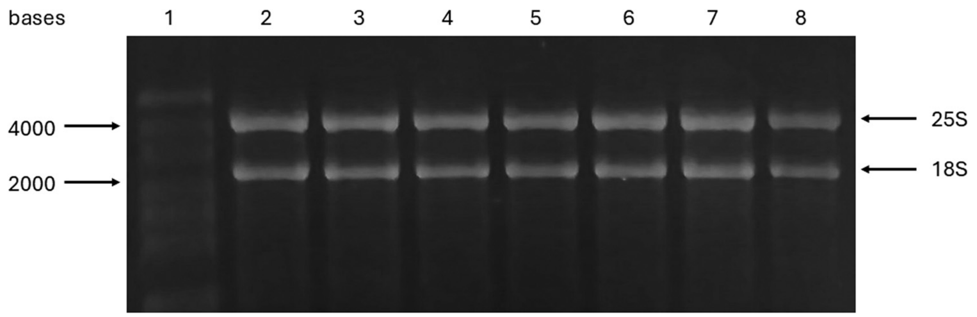

Figure S1: Agarose gel electrophoresis of total RNA isolated from *N. glabratus.* Approximately 1 µg of total RNA was loaded into each gel lane. The gel was stained with ethidium bromide.. Lane 1 – RNA Ladder; lane 2 contains RNA from cells grown in YPD medium for 4 h; lane 3 from YPD medium for 18 h; lane 4 from YPDA medium for 4 h; lane 5 from YPDA medium for 18 h; lane 6 from RPMI 1640 medium for 4 h; lane 7 from RPMI 1640 medium for 18 h; and lane 8 from cells grown in VS medium for 18 h.
